# Computational epigenomics: challenges and opportunities

**DOI:** 10.3389/fgene.2015.00088

**Published:** 2015-03-05

**Authors:** Mark D. Robinson, Mattia Pelizzola

**Affiliations:** ^1^Institute of Molecular Life Sciences, University of ZurichZurich, Switzerland; ^2^SIB Swiss Institute of Bioinformatics, University of ZurichZurich, Switzerland; ^3^Computational Epigenomics, Center for Genomic Science of IIT@SEMM, Istituto Italiano di TecnologiaMilano, Italy

**Keywords:** epigenomics, histone mark, DNA methylation, ChIP-seq, epigenetic code

The field of epigenetics is undoubtedly attracting immense interest with countless studies in various areas of investigation; (see Rivera and Ren, 2013) for a review on the state of the art for human epigenomics. From the computational point of view and the characteristics of the generated data, epigenomics is a very complex field, for two main reasons. First, epigenetics encompasses a multi-layered set of regulatory cues that act coordinately and possibly in a combinatorial way to control fundamental biological processes, such as the output of gene expression programs. Second, profiling techniques based on high-throughput sequencing are widely adopted in this field, generating comprehensive yet complex and massive genome-wide datasets. As a result, the contribution of scientists with computational skills (computer scientists, statisticians, physicists and computational biologists) is considered an essential component of research institutes investing in this research field (Bock and Lengauer, 2008).

In this Research Topic, we collected a number of contributions in the field of computational epigenomics covering three main research areas: (i) computational analyses tackling important issues closely related to the experimental method used to generate epigenetic data (Flensburg et al., 2014; Ji et al., 2014; Mensaert et al., 2015), (ii) computational approaches useful to overcome pitfalls associated to the analysis of a given epigenetic layer (Barozzi et al., 2014; Cairns et al., 2014; Robinson et al., 2014), and (iii) studies on the integration of multiple epigenetic layers (de Pretis and Pelizzola, 2014; Fejes et al., 2014; Osella et al., 2014).

Computational tools developed for the analysis of specific epigenetic data types, including DNA methylation and ChIP-seq of histone post-translational modifications (so-called “marks”), have to deal with the biases originated directly from the experimental methodology. In the case of profiling DNA methylation, various approaches based on sequencing are available, depending on the desired tradeoff between cost, coverage and data resolution. In some cases, a non-trivial subset of the DNA fragments sequenced in MBD-seq experiments, based on affinity purification through a methyl-CpG binding protein, could not be assigned to the expected reference genome. It was then shown how it is possible to assess this unanticipated proportion of unmapped reads to profile methylated viral sequences, which can be particularly relevant in certain studies (e.g., oncoviruses; Mensaert et al., 2015). On the other hand, reads from methylated DNA were shown to be over-represented in data from whole-genome bisulfite sequencing experiments. The technical reasons for this bias and the necessity of developing computational methods for correcting this issue, especially when interested in allelic methylation, were explored (Ji et al., 2014). Finally, regarding the analysis of ChIP-seq data, computational methods were shown to be helpful in clarifying how to generate reference samples necessary for the identification of enriched genomic regions. Specifically, the effect of using pull-down of the whole histone H3 or the more common input sample (whole-cell extract) were compared, showing how this choice had negligible impact on the resulting computational results (Flensburg et al., 2014).

Various computational methods have been developed for the analysis of different epigenetic data types, yet it remains difficult to understand the relative merits and performance of all the available approaches. Trying to guide on the identification of the best-suited method, a number of contributions in this Research Topic focused on the comparison between computational methods and discussion to contrast the available analysis strategies. Regarding DNA methylation data, a number of methods developed for the identification of differentially methylated bases or regions were compared, while discussing the importance of experimental design, and confounders such as batch effects and cell type composition (Robinson et al., 2014); this is a very active field, evidenced by new tools emerging, such as DMRcate (Peters et al., 2015) and M3D (Mayo et al., 2014) and also highlighting the need to constantly update performance comparisons. Touching on a different data type, chromatin accessibility, various methods for the identification of footprints in DNase-seq were discussed and compared using ENCODE data (Barozzi et al., 2014). Accessibility only reveals information about presumed activity, but is commonly chosen since it is complimentary to the analysis of specific epigenetic marks and provides a list of putative regulatory proteins that bind open chromatin regions. Finally, tackling the issue of the statistical modeling of read counts for ChIP-seq data, various alternatives were discussed and a method based on double Negative Binomial (i.e., Poisson distributed counts with a mixture of two gamma-distributed rates) was proposed (Cairns et al., 2014).

While the experimental methods and the computational analysis of individual data types are compared and perfected, scientists are investigating how to make connections between the various epigenetic layers that are surveyed. It is now clear that patterns of DNA methylation and histone marks are established, maintained and have effect through a machinery that is influenced by the crosstalk between these layers, and their interplay with binding of regulatory proteins, chromatin accessibility and 3D conformation. In other words, the joint analysis of multiple epigenetic layers through data integration methods (Ritchie et al., 2015) is considered the key to comprehend how epigenetic information contributes controlling complex regulatory processes. In this series of articles, computational and experimental methods for the integrative analysis of epigenetic marks are discussed and proposed. Double-negative feedback loops, where a microRNA is inhibited by an epigenetic regulator while being epigenetically controlled by the same regulator, are considered and shown to exhibit properties that are well suited for circuits involved in cell fate transitions (Osella et al., 2014). In the context of data integration and visualization, an online platform (DaVIE) was developed based on a database of DNA methylation experiments. This tool allows navigating through multiple DNA methylation experiments and integrating different data types, including ChIP-seq data (Fejes et al., 2014). Finally, recent and past evidence in favor of the notion of epigenetic code are discussed, and computational and experimental strategies are proposed that can be instrumental to further investigating how different epigenetic layers and marks are interconnected (de Pretis and Pelizzola, 2014).

Altogether, this series of articles provides a comprehensive glance at the emerging field of computational epigenomics. This research area brings to the field of epigenetics a set of tools that were initially developed in the field of genomics. At the same time, computational epigenetics is showing its maturity toward closing the circle between the genome and the epigenome, revealing how regulatory layers are interconnected and highlighting the need to jointly consider epigenetic phenomenon to explain complex transcriptional responses.

## Conflict of interest statement

The authors declare that the research was conducted in the absence of any commercial or financial relationships that could be construed as a potential conflict of interest.
